# Down-regulated miR-9 and miR-433 in human gastric carcinoma

**DOI:** 10.1186/1756-9966-28-82

**Published:** 2009-06-16

**Authors:** Hongchun Luo, Hongbin Zhang, Zhenzhen Zhang, Xia Zhang, Bo Ning, Jinjun Guo, Na Nie, Bo Liu, Xiaoling Wu

**Affiliations:** 1 Department of Gastroenterology, The Second Affiliated Hospital, Chongqing Medical University, Chongqing 400010, PR China; 2 Department of Hematology, The First Affiliated Hospital, Chongqing Medical University, Chongqing 400016, PR China; 3 The Key Laboratory of Molecular Biology of Infectious Diseases of Ministry of Education, Viral Hepatitis research Institute, Chongqing Medical University, Chongqing 400016, PR China; 4 Department of Laboratory Medicine, Chongqing Medical University, Chongqing 400016, PR China

## Abstract

**Background:**

MircoRNAs(miRNAs) are short, endogenously non-coding RNAs. The abnormal expression of miRNAs may be valuable for the diagnosis and treatment of tumors.

**Methods:**

To screening the special miRNAs in gastric carcinoma, expression level of miRNAs in gastric carcinoma and normal gaster samples were detected by miRNA gene chip. Then, the expressions of miR-9 and miR-433 in gastric carcinoma tissue and SGC7901 cell line were validated by qRT-PCR. GRB2 and RAB34, targets of miR-433 and miR-9 respectively, were detected by Western blot.

**Results:**

We found 19 miRNAs and 7 miRNAs were down-regulated and up-regulated respectively. Compared with normal gaster samples, our data showed that miR-9 and miR-433 were down-regulated in gastric carcinoma. Meanwhile, we also found that miR-433 and miR-9 regulated the expression levels of GRB2 and RAB34 respectively.

**Conclusion:**

Our data show miR-9 and miR-433 was down-regulated in gastric carcinoma. The targets of miR-433 and miR-9 were tumor-associated proteins GRB2 and RAB34 respectively. This result provided the related information of miRNAs in gastric carcinoma.

## Background

Gastric cancer is the fourth most common malignancy and the second cause of death [1]. Many studies indicated that gastric carcinoma is a polygenic disease with multistep processes for the abnormal development of many related genes [2]. However, the regulatory mechanism involved in the development of canceration is still not well understood.

Recently, researchers have found a new class of short, endogenously non-coding RNAs called microRNAs(miRNAs) in animals and plants [3-5]. They regulate the expression of protein-coding genes via degrading or inhibiting the translation of the targeted mRNAs[6]. Accumulated evidences demonstrated miRNAs play important role in carcinogenesis. Xiao indicated miRNA-106a (miR-106a) had oncogenic activity in humans. The level of miR-106a in cancer tissues was significantly higher than that in non-tumor tissues expression [7]. Another paper showed that restoration of tumor suppressor miR-34 inhibits human p53-mutant gastric cancer tumorspheres [8]. Together, these observations suggest the possible existence of cancer-specific miRNAs. For this reason, miRNAs expression profiling has been investigated in different kinds of cancer to identify cancer-specific miRNAs [9-13]. In the present study, we detected the expression profiling of 328 miRNAs in 2 cell lines, 24 gastric cancer samples and 3 normal gastric tissue samples, revealing the miRNA characteristics of gastric cancer. Furthermore, our data suggested significantly down-regulated miR-433 and miR-9, which were considered as the modulator of GRB2 and RAB34 respectively. GRB2 and RAB34 were involved in the molecular pathogenesis of gastric cancer.

## Methods

### Gastric tissues and cell lines culture

All human gastric tissue samples including 3 normal gastric tissues and 24 malignant tissues (2 in early phase and 22 in late phase of gastric cancer) were obtained from General surgery dept. of the First and Second Affiliated Hospital of Chongqing Medical University (Chongqing, China). All the patients signed the informed consent. The tissues were stored in liquid nitrogen after removing from patients. Gastric cancer cell SGC7901 was donated by Viral Hepatitis Research Institute of Chongqing Medical University (Chongqing, China). Gastric cell line GES-1 was purchased from Cancer Institute and Hospital of Chinese Academy of Medical Sciences (Beijing, China). Both cell lines were cultured in serum-free Roswell Park Memorial Institute (RPMI) 1640 medium supplemented with 10% low-endotoxin FCS.

### Total RNA preparation

Total RNAs were extracted from malignant tissues, normal gastric tissues, SGC7901 and GES-1 respectively by using Trizol in accordance with the manufacturer's instructions. The total RNAs were quantified by ultraviolet spectrophotometer at 260 nm.

### miRNA microarray hybridization

Total 33 miRNA microarrays were used to examine miRNA expression profiling. 3 miRNA microarrays were used for 3 normal gastric tissues, 24 miRNA microarrays were used for 24 malignant tissues, and 6 for SGC7901 and GES-1 cell lines. 5 μg total RNAs from each sample were used for miRNA labeling. Then, miRNA array hybridizations were performed on miRNA microarray. A GenePix 4000B scanner (Axon Instruments) was employed to detect hybridization signals via streptavidin-Alexa Fluor 647 conjugation. Images were quantified by the GenePix Pro 6.0(Axon Instruments).

### Reverse transcription

The total RNAs were reverse transcribed to synthesize cDNA. The RT Primers were designed by Primer 5.0 software and shown in Table 1. The 20 μl reaction system included 2 μl dNTPs (HyTest Ltd), 2 μl 10× RT Buffer (Epicentre), 1 μl RTspecific primer, 1 μg Total RNA, 2 μl M-MLV reverse transcriptase (Epicentre), 0.3 μl RNase inhibitor (Epicentre) and nucleas-free ddH_2_O. The reaction was performed at 16°C for 30 min, 42°C for 42 min followed by 85°C for 5 min. The process was performed in Gene Amp PCR System 9700 (Applied Biosystems). The reverse transcription products were stored at -20°C for use.

**Table 1:** Reverse transcription primers

Gene name	RT primer
U6	5'CGTTCACGAATTTGCGTGTCAT3'
hsa-miR-9	5'GTCGTATCCAGTGCGTGTCGTGGAGTCGGCAATTGCACTGGATACGACTCATACAG3'
hsa-miR-433	5'GTCGTATCCAGTGCGTGTCGTGGAGTCGGCAATTGCACTGGATACGACTCACACCG3'

### Quantitative Real-time PCR

The expressions of miR-9 and miR-433 in 29 samples were identified by qRT-PCR. The interested miRNAs and an interior reference *U6 *were run in Rotor-Gene 3000 Real-time PCR (Corbett Research). Real-time PCR primers were shown in Table 2. 25 μl PCR mixture included 2.5 μl dNTPs (HyTest Ltd), 2.5 μl 10 × PCR Buffer (Promega), 1.5 μl MgCl_2 _liquor (Promega), 1 unit *Taq *polymerase (Promega), SybergreenI (Invitrogen) final concentration 0.25×, 1 μl PCR specific primer forward and reverse, 1 μl reverse transcription product and nucleas-free water. The reactions were performed at 95°C for 5 min, then followed by 40 cycles of 95°C for 10 s and 60°C for 1 min. The expression of miRNA was measured by Ct(threshold cycle). The Ct represented the fractional cycle number when the fluorescence of each sample passed the fixed threshold. The ΔΔCt method determined miRNA expression level. The change was generated using the equation: 2 ^-ΔΔCT^.

**Table 2:** Quantitative Real-time PCR primers

Gene name	Primer sequence	Anneal temperature(°C)	Product length (bp)
U6	F:5'GCTTCGGCAGCACATATACTAAAAT3'	60	89
	R:5'CGCTTCACGAATTTGCGTGTCAT3'		
hsa-miR-9	F:5'GGGTCTTTGGTTATCTAGC3'	60	64
	R:5'TGCGTGTCGTGGAGTC3'		
hsa-miR-433	F:5'GGATCATGATGGGCTCCT3'	60	63
	R:5'CAGTGCGTGTCGTGGAGT3'		

### Prediction and clone of miRNA target

Predicted targets of miRNA were determined by the algorithms: TargetScan, PicTar, and miRanda. In this way, we found that RAB34 and GRB2 were the predicted targets of miR-9 and miR-433 respectively. The 3'-UTR target sites of the human RAB34 and GRB2 were synthesized and cloned in the downstream of the luciferase gene of pGL3-control. The plasmids including junction fragments of norientation were screened by PCR. Amplification primers of Plasmid containing miR-9 target (about 430 bp products): forward (5'-TGGACGAAGTACCGAAAGGT-3') and reverse (5'-GGCACAGTGAGAGGCTGGAATCATTAAGCATCCTCAAAC); The Amplification primers of Plasmid containing miR-433 target (about 580 bp products): forward (5'-TGGGAGTCTCCCTCCGACTCCAGATATGAA-3') and reverse (5'-CACTGCATTCTAGTTGTGGT-3'). Both plasmids were identified by *XbaI *digestion and electrophoresis. The sequenced plasmids were named pGL3-miR-9 and pGL3-miR-433 and used for SGC7901 cell transfection.

### Transfection and assay of luciferase activity

To examine the luciferase activity, 4 groups were set up for miR-9 and miR433. Respectively, ①SGC7901 (blank control), ②pGL3, ③pGL3-miR-9, ④hsa-miR-9 (Takara Co., Ltd. Danian, China)+ pGL3-miR-9 for miR-9 and ①SGC7901 (blank control), ②pGL3, ③pGL3-miR-433, ④hsa-miR-433 (Takara Co., Ltd. Danian, China) + pGL3-miR-433 for miR-433. SGC7901 cells were seeded in 6-well plates for 24 h before the transfection. When the cell were 50%~60% confluence, 2 μg of plasmids were transfect in each group. Transfection was performed using Lipofectamine 2000 (Invitrogen)according to the manufacturer's procedure. After the 48 h transfection, luciferase activity was assayed and analyzed by relative light unit (RLU).

### Western blot analysis

To evaluate regulation of RAB34 and GRB2 by miR-9 and miR-433, SGC7901 was transfected with miR-9 and miR-433 in a 6-well plate according to manufacturer's procedure. For both miR-9 and miR-433, there were three groups including ①control group; ②group 1: 50 pmol of miR-9 or miR-433 was transfected; ③group 2: 100 pmol of miR-9 or miR-433 was transfected. After 48 h transfection, the cells were harvested and total protein and total RNA were extracted. RAB34 and GRB2 expression levels were detected by Western blot. MiR-9 and miR-433 level were mesured by qRT-PCR respectively.

### Statistics and presentation of data

All data are expressed as means ± standard deviation. Each experiment was repeated at least 6 times. The *t *test was used to examine the differences between groups. A p value of less than 0.05 was considered as significance.

## Results

### Expressive characteristics of miRNA in gastric cancer tissues and cell lines

A conventional microarray platform was used to evaluate miRNA expression profiling in 3 normal gastric tissues, 24 malignant tissues, SGC7901 and GES-1 cell lines. Compared with that in the normal gastric samples, 26 miRNAs expressed abnormally in gastric carcinoma samples. 19 miRNAs of these were down-regulated (the expression in the normal group was more than twice as high as in the carcinoma group). 7 miRNAs were up-regulated (the expression in the carcinoma group was more than twice as high as in the normal group). The differentially expressive miRNAs were listed in Table 3.

**Table 3:** miRNAs differential expression in gastric cancer samples compared with the normal samples

Down-regulation (19)	*P *Value	Up-regulation (7)	*P *Value
miR-9	0.0073	miR-518b	0.009
miR-433	0.0041	miR-26b	0.0147
miR-490	0.0142	miR-212	0.0329
miR-155	0.021	miR-320	0.0179
miR-188	0.019	miR-409-3b	0.0352
miR-630	0.024	miR-30a-5b	0.0164
miR-503	0.0102	miR-379	0.0158
miR-611	0.0151		
miR-545	0.0241		
miR-567	0.0173		
miR-575	0.0109		
miR-197	0.024		
miR-649	0.0157		
miR-19b	0.017		
miR-338	0.0184		
miR-383	0.0267		
miR-652	0.0183		
miR-551a	0.0166		
miR-370	0.0112		

### Detection of miR-433 and miR-9 expression by Quantitative Real-time PCR

**Figure 1:**
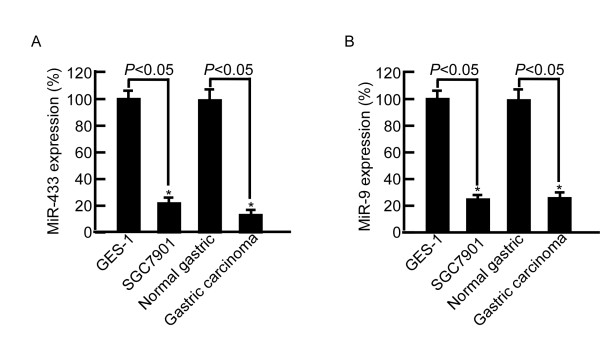
**MiR-433 and miR-9 expression in normal gastric tissues, 24 malignant tissues, SGC7901 and GES-1 cell lines**. A, miR-433 was down-regulated 83% in the carcinoma tissues compared with normal gastric tissues and down-regulated 77.3% (P < 0.05) in SGC7901 compared with GES-1 cell lines. B, miR-9 was down-regulated 75% in carcinoma tissues compared with normal gastric tissues and down-regulated 76.2% (*P *< 0.05) in SGC7901 compared with GES-1 cell lines.

MiR-433 and miR-9 were remarkably down-regulated by microarray analysis in the carcinoma samples. qRT-PCR was used to detect the expressive level of miR-433 and miR-9 in 3 normal gastric tissues, 24 malignant tissues, SGC7901 and GES-1 cell lines. We found that miR-433 was down-regulated 83% in the carcinoma tissues compared with normal gastric tissues. MiR-433 was down-regulated 77.3% (*P *< 0.05) in SGC7901 compared with GES-1 cell lines (Figure 1A). MiR-9 was down-regulated 75% in carcinoma tissues compared with normal gastric tissues. MiR-9 was down-regulated 76.2% (*P *< 0.05) in SGC7901 compared with GES-1 cell lines (Figure 1B). The results were consistent to the microarray analysis.

### Identification of miR-9 and miR-433 targets

**Figure 2:**
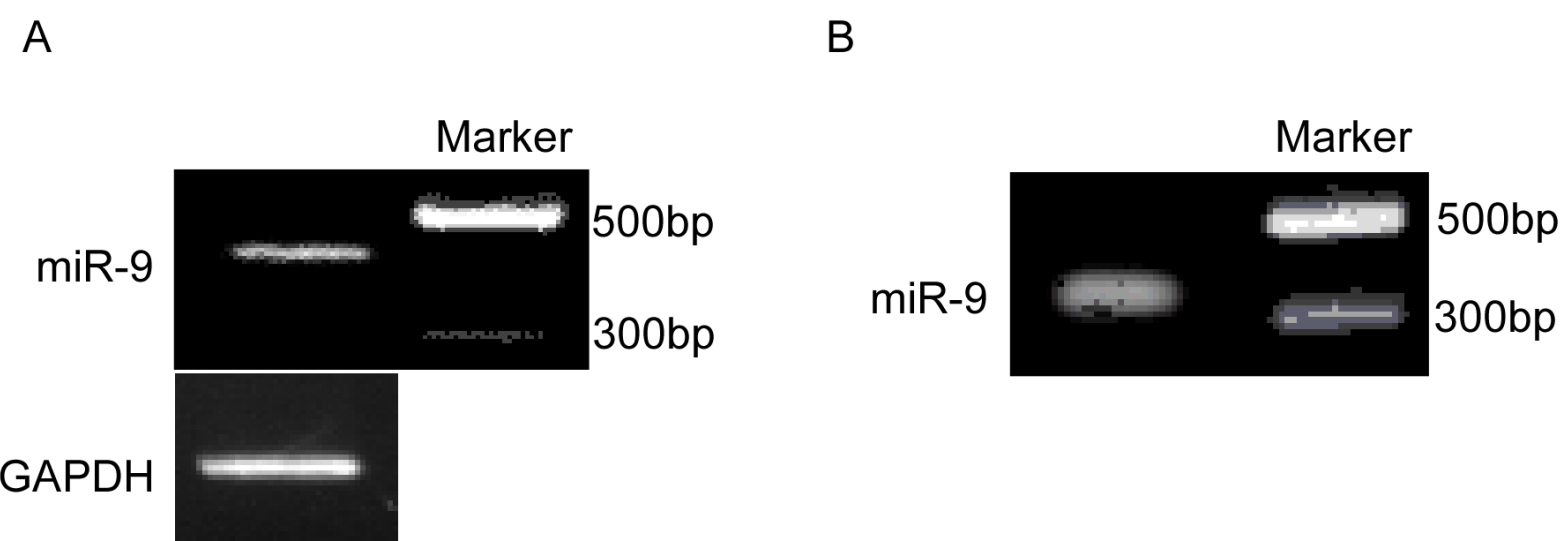
**Cloning of miR-9 target gene**. A, identification of junction fragment of norientation. There was a 430 bp fragment, which demonstrated that the fragment was norientation. B, junction fragment digested by XbaI. The 360 bp fragment was destination fragment.

**Figure 3:**
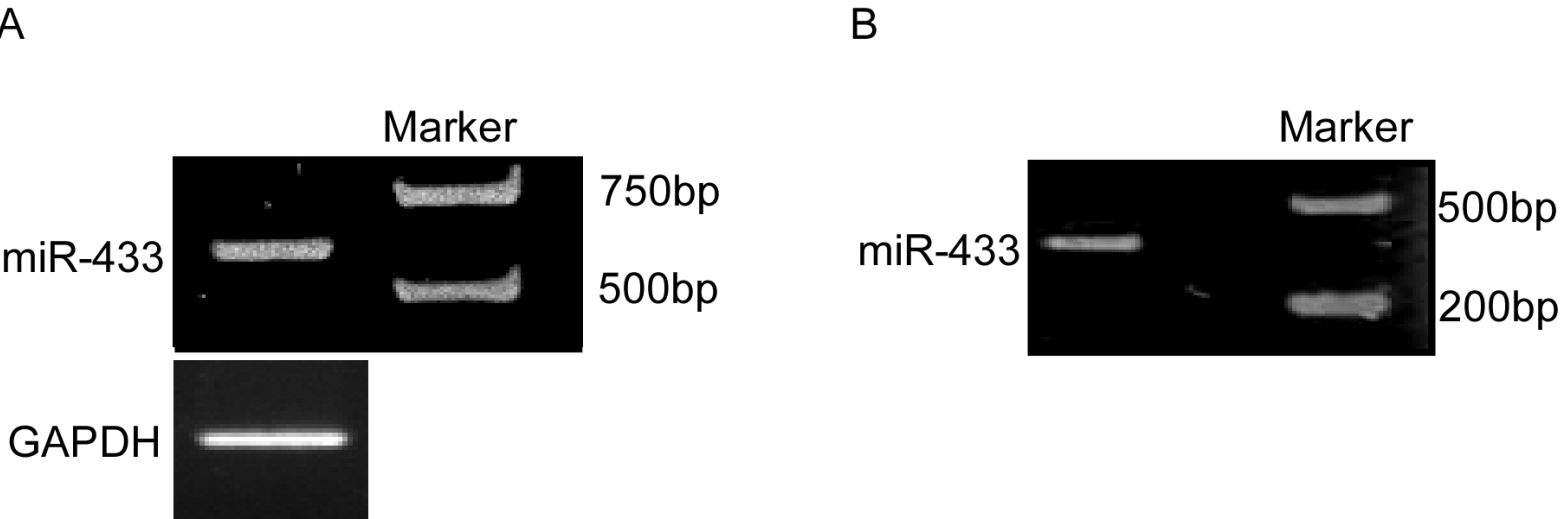
**Cloning of miR-433 target gene**. A, identification of junction fragment of norientation. There was a 580 bp fragment, which demonstrated that the fragment was norientation. B, junction fragment digested by XbaI. The 360 bp fragment was destination fragment.

We were further interested in miRNA-regulated gene targets, which enabled us to understand miRNA functions. To explain the potential roles of miR-9 and miR-433 in carcinogenesis, we predicted the targets of miR-9 and miR-433 via the algorithms: TargetScan, PicTar, and miRanda. To confirm whether the predicted targets of miR-9 and miR-433 were responsible for their regulation, the presumed target sites were cloned and inserted at the downstream of the luciferase gene of pGL3. Direction of junction fragments was identified and plasmids including junction fragments of norientation were chose. In Figure (2A), we found a 430 bp fragment, and in Figure (3A), we found a 580 bp fragment. The results were consistent to the amplification of pGL3-control and junction fragments sequences, which demonstrated that the fragments were norientation. *XbaI *was used to digest the junction fragments, then, we did electrophoresis. In Figure (2B) and Figure (3B), 360 bp fragments were objective fragments. The fragment was sequenced and inserted into plasmids.

**Figure 4:**
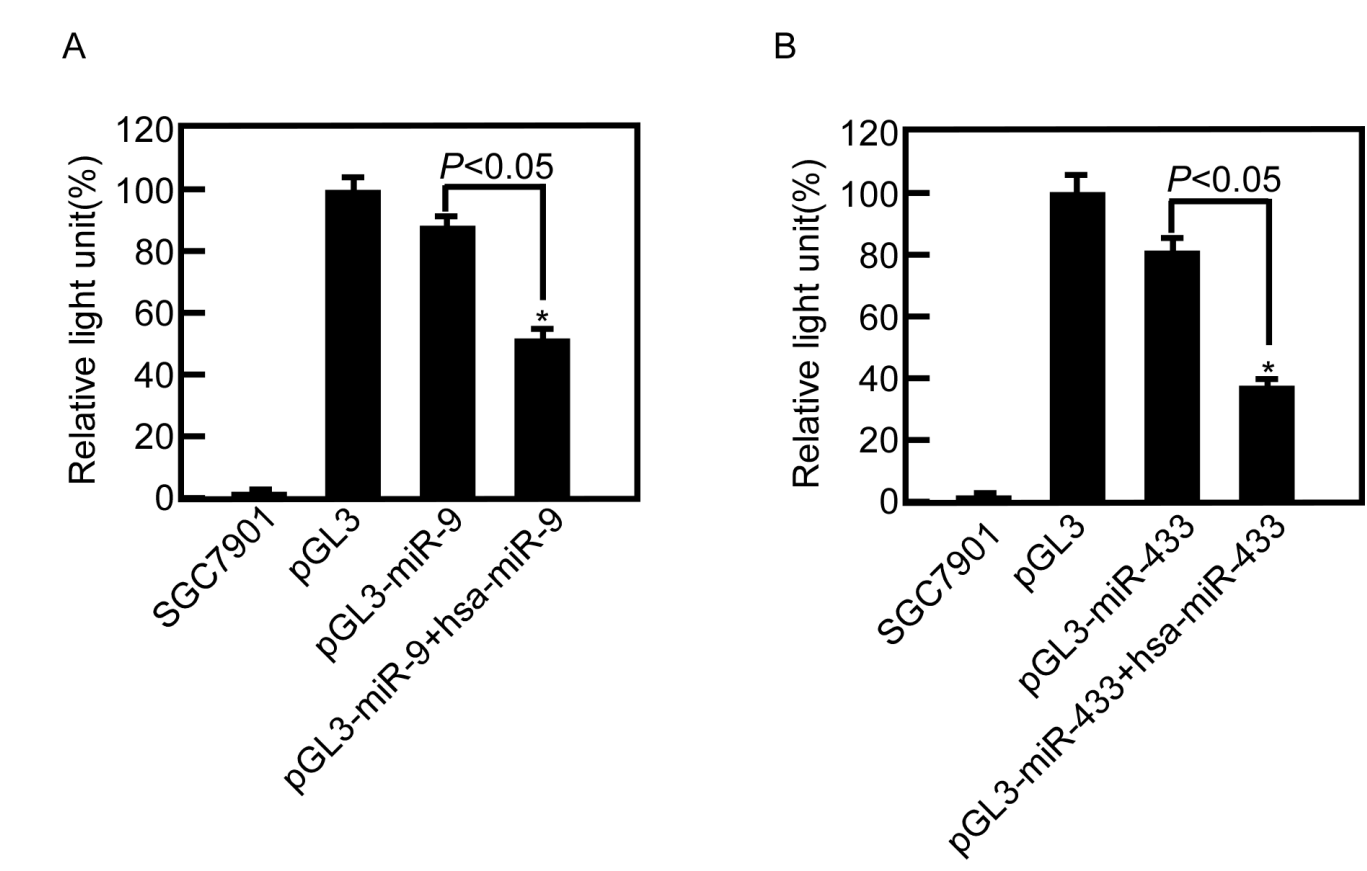
**miR-9 and miR-433 down regulated luciferase activity of RAB34 and GRB2**. A, miR-9 regulated luciferase activity by integrating the binding site in the 3'-UTR of RAB34. Luciferase activity of SGC7901 cotransfected pGL3-miR-9 and hsa-miR-9 decreased 50% compared with pGL3-miR-9 (P < 0.05). B, miR-433 regulated luciferase activity by integrating the binding site in the 3'-UTR of GRB2. Luciferase activity of SGC7901 cotransfected pGL3-miR-433 and hsa-miR-433 decreased 54% compared with pGL3-miR-433 (*P *< 0.05).

We measured luciferase activity and the relative light unit (RLU) at 48 h after the transfection. Luciferase activity of cells cotransfected pGL3-miR-9 and hsa-miR-9 decreased 50% compared with pGL3-miR-9 (*P *< 0.05) (Figure 4A). Luciferase activity of cells cotransfected pGL3-miR-433 and hsa-miR-433 decreased by 54% compared with pGL3-miR-433 (*P *< 0.05) (Figure 4B).

**Figure 5:**
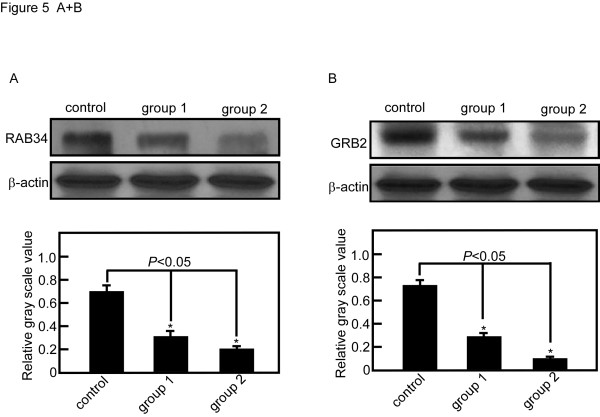
**miR-9 and miR-433 down regulated RAB34 and GRB2 expression in SGC7901 cell line**. A, RAB34 decreased 45% and 72% compared with control group after 50 pmol (group 1) and 100 pmol (group 2) hsa-miR-9 transfection. Relative gray scale value was compared with β-actin. B, GRB2 decreased 53% and 89% compared with control group after 50 pmol (group 1) and 100 pmo l (group 2) hsa-miR-433 transfection. Relative gray scale value was compared with β-actin.

**Figure 6:**
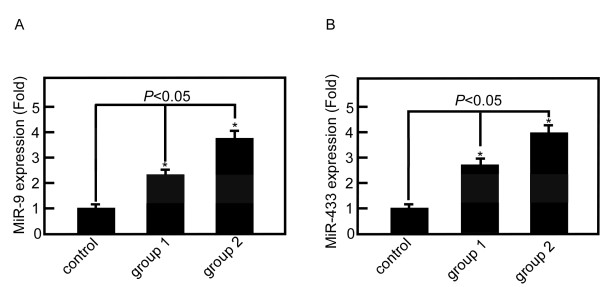
**MiR-9 and miR-433 increased after hsa-miR-9 and hsa-miR-433 transfection**. A, miR-9 level increased 1.3-fold and 2.8-fold respectively after 50 pmol (group 1) and 100 pmol (group 2) hsa-miR-9 transfection. B, miR-433 level increased 1.6-fold and 3.0-fold respectively after 50 pmol (group1) and 100 pmol (group 2) hsa-miR-433 transfection. (*P *< 0.05).

The expression level of RAB34 and GRB2 were measured after miR-9 or miR-433 were transfected into SGC7901. The expression of RAB34 decreased 45% in group 1 and 72% in group 2 compared with control group (P < 0.05) (Figure 5A). The expression of GRB2 decreased 53% in group 1 and 89% in group 2 compared with control group (*P *< 0.05) (Figure 5B). Meanwhile, we measured the level of miR-9 and miR-433 by qRT-PCR. MiR-9 level increased 1.3-fold and 2.8-fold respectively in group 1 and 2 compared with control group (*P *< 0.05) (Figure 6A). MiR-433 level increased1.6-fold and 3.0-fold in group 1 and 2 compared with control group (*P *< 0.05) (Figure 6B).

## Discussion

Recent researches indicated some miRNAs were down-regulated in human malignancies. Meanwhile, these miRNAs could be used to classify histotypes of tumors, distinguish cancer tissue from normal tissue [14-17]. In present study, the expression of 328 miRNAs in 3 normal gastric tissues, 24 malignant tissues, SGC7901 and GES-1 were detected to screen specific miRNAs markers for gastric carcinoma. 26 miRNAs were found expression abnormally in gastric carcinoma samples. 19 miRNAs was down-regulated and 7 miRNAs were up-regulated. The number of the down-regulated miRNAs in carcinoma samples was more than that of the up-regulated ones in the past studies on tumor related miRNAs [9,14], which was consistent to our former results. The absence of mechanism of miRNAs maturation might explain the general down-regulation of miRNAs in tumors [18]. However, miRNAs maturation was activated in some studies [19], which was the reason for the unclear role of miRNAs maturation procession in tumorigeness.

Although different types of tumors may have the same miRNAs markers, there are specific miRNAs in tumors from different cellular origins [20]. In this study, majority of the differentially expressive miRNAs have not been reported in other tumors, especially miR-433 and miR-9. Both of them were down-regulated significantly in gastric carcinoma tissue and SGC7901 cell line, suggesting they might be the special markers for gastric carcinoma.

The differential expressions of miRNAs suggest miRNAs may be involved in the genesis and development of tumor. Up to now, the relation between down-regulated miRNAs and tumorigenesis was not well understood. Although bioinformatics could be used to predict the targets of miRNAs, these targets still need to be confirmed by experiment. Studies have confirmed that miRNAs could regulate the expressions of oncogenes. For example, miRNAs of let-7 family could regulate 3 members of RAS oncogene family [21] and miR-15a/miR-16-1 could regulate BCL2 [22], which supported the down-regulated miRNAs were involved in tumors nosogenesis. MiR-9 and miR-433 were found down-regulated significantly in gastric carcinoma samples, suggesting they might play important roles in the cancerigenic process. Meanwhile, we confirmed that RAB34, GRB2 were down regulated by miR-9 and miR-433 respectively, which revealed the potential mechanism for gastric carcinoma genesis. RAB34 is a member of RAS oncogene family. It is a guanosine triphosphatase (GTPases) which can regulate budding, junction and fusion of vesicle in exocytosis and endocytosis pathway [23]. GRB2, an adaptor protein, is a growth factor binding protein. GRB2 binds to the phosphorated tyrosine residue of the receptor via SH2 domain after receptor tyrosine kinase (RTK) is activated. Meanwhile, GRB2 binds to proline enrichment region of Sos protein via its SH3 domain and formed receptor-GRB2-Sos signal transduction complex. Sos stimulates proximal Ras to release GDP and bind to GTP, which leads to the activation. Once activated, Ras activates various signal transduction proteins in different signal pathways of the downstream. Mitogen-activate-protein kinases (MAPKs) system is an important pathway among them. MAPK plays an important role in cell growth, proliferation, differentiation. Meanwhile, it is involved in cellular stress reaction. In this study, we found the expressive levels of miR-433 and miR-9 was significantly down-regulated in gastric cancer tissues and SGC7901. MiRNAs also can silence gene. The down-regulation of miR-433 and miR-9 attenuated the gene silencing, which activated GRB2 and RAB34.

In summary, we found miRNAs expressions profiling in human gastric carcinoma, and focused on the screen and identification of targets of the abnormally expressive miRNAs. Our results showed miR-433 and miR-9 was significantly down-regulated and might be used as a marker for the advanced gastric carcinoma. In addition, we also found miR-433 and miR-9 targeted GRB2 and RAB34, which was favorable for explaining carcinogenesis pathway mediated by miRNAs and screening the therapeutic targets. Some researchers have found that successive short RNAs injection could affect liver effectively in vivo [24,25], which established a good model for the development of miRNA-based approach of gene therapy. Our results show the differentially expressive miRNAs in gastric carcinoma, which will provide related data for molecular targeted therapy based on miRNAs.

## Competing interests

The authors declare that they have no competing interests.

## Authors' contributions

HL performed Quantitative Real-time PCR, clone of miRNA target, transfection and assay of luciferase activity, and drafted the manuscript. HZ performed Western blot analysis. ZZ performed miRNA microarray hybridization. XZ performed total RNA preparation and reverse transcription. BN conceived of the idea and provided helpful comments. JG analyzed data and helped write the manuscript. NN purchased and cultured cell lines. BL collected tissue specimens and clinical records. XW conceived of the study and guided the biochemical experiments. All authors read and approved the final manuscript.
